# Gender dysphoria and neurodiversity: understanding the intersections

**DOI:** 10.1192/j.eurpsy.2026.10371

**Published:** 2026-07-31

**Authors:** D. Pauli

**Affiliations:** Child and Adolescent Psychiatry, Psychiatric University Hospital Zurich, Zurich, Switzerland

## Abstract

**Abstract:**

Treatment units for gender dysphoria show an increased number of people with neurodiversity compared to the general population. Similarly, outpatient units for autism show a high percentage of people who also suffer from gender dysphoria. The causes for this clustering are unknown, altohugh it has now been etablished as a fact in research. Studies show that people with both conditions experience particularly high levels of psychological stress and concomitant psychiatric disorders. Treating people with neurodiversity and gender dysphoria requires special expertise in both areas. Surveys of experts using the Delphi method resulted in guidelines about how specific features need to be taken into account, and how both condition sshould be addressed at the same tine. it is ethically unacceptable to deny people with neurodiversity access to gender reassignment mesures. However, they often need more support during the assassement and transition process,

**Disclosure of Interest:**

None Declared.

